# Comparative efficacy of AHU-377, a potent neprilysin inhibitor, in two rat models of volume-dependent hypertension

**DOI:** 10.1186/1471-2210-11-S1-P33

**Published:** 2011-08-01

**Authors:** Laxminarayan G Hegde, Cecile Yu, Cheruvu Madhavi, Russell Araki, Jennifer Villarreal, Glenmar Obedencio, Anne Kanta, Erik Sandvick, Craig Hill, Kevin Dement, Uwe Klein, Donavon McConn, William Martin, Sharath S Hegde

**Affiliations:** 1 Department of Pharmacology,Theravance Inc., South San Francisco, CA-94080, USA; 2 Department of Molecular & Cellular Biology, Theravance Inc., South San Francisco, CA-94080, USA; 3 Department of Drug Metabolism & Pharma cokinetics, Theravance Inc., South San Francisco, CA-94080, USA

## Background

Progressive hypertension and deteriorating renal function are hallmarks of cardio-renal syndromes. Historically, the deoxycorticosterone acetate (DOCA)-salt model has been used to assess the therapeutic potential of antihypertensives in the setting of low renin and volume-dependent hypertension. Dahl-salt-sensitive (Dahl-SS) rats represent a genetic model of volume-dependent hypertension and possess similar features to the DOCA model, including higher natriuretic peptide tone. Neprilysin inhibitors (NEPi) prevent the degradation of natriuretic peptides and activate cGMP signalling pathways that regulate volume and blood pressure. Antihypertensive effects of NEPi have been demonstrated in both DOCA and Dahl-SS models; however, the relative efficacy of NEPi in these two models has not been studied directly. Here, we characterized the natriuretic peptide tone in DOCA and Dahl-SS rats and compared antihypertensive effects of a NEPi, (AHU-377).

## Methods

We determined the relationship between atrial natriuretic peptide (ANP) and blood pressure in anesthetized, normotensive rats. We studied the relationship between NEP inhibition and elevation of plasma cGMP evoked by ANP in the absence and presence of AHU-377, an ester prodrug of LBQ657 and a component of LCZ696 [1,2]. Finally, using telemetry, we assessed the antihypertensive effects of AHU-377 in conscious Dahl-SS and DOCA-salt models of hypertension.

## Results

**Figure 1:**
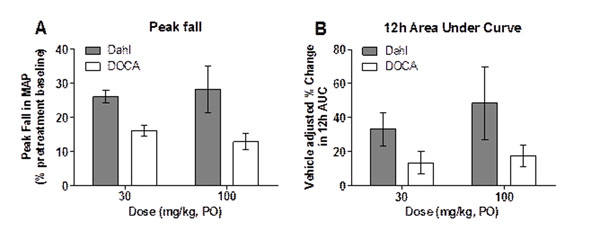
Comparison of peak fall in MAP (relative to baseline; A) and 12 hour vehicle adjusted AUC _MAP_ (B) following oral administration of AHU-377 (30 and100 mg/kg, PO) in conscious Dahl-SS and DOCA-salt hypertensive rats (n=4-7 per treatment group).

ANP reduced mean arterial pressure (MAP) by 4, 17, 29 and 34 mmHg at doses of 0.1, 1, 10 and 100 µg/kg IV, respectively. ANP also promoted diuresis and increased plasma and urinary cGMP. In normotensive rats, pretreatment with AHU-377 (3, 10 and 30 mg/kg, PO.) augmented ANP-evoked plasma cGMP levels by 2.4, 3.3 and 4.0 fold, respectively (4h AUC compared to vehicle). In Dahl-SS rats, AHU-377 (30 and 100 mg/kg, PO) produced a dose-dependent antihypertensive effect (Figure 1). By contrast, AHU-377 exerted only a modest reduction in MAP in the DOCA-salt hypertensive rats, despite achieving estimated neprilysin enzyme occupancy of >95% at highest dose tested (100 mg/kg, PO).

## Conclusion

These studies confirm the role of the natriuretic peptide system in blood pressure control. AHU-377 enhances the tone of the natriuretic peptide system and exerts significant antihypertensive effects. These effects were more pronounced in Dahl-SS than in DOCA-salt rats. The apparent higher sensitivity of Dahl-SS rats to neprilysin inhibition suggest that this model could provide a better method for testing the antihypertensive efficacy of neprilysin inhibitors.
